# Urinary citrate excretion in healthy children depends on age and gender

**DOI:** 10.1007/s00467-014-2806-7

**Published:** 2014-04-03

**Authors:** Jan K. Kirejczyk, Tadeusz Porowski, Jerzy Konstantynowicz, Agata Kozerska, Andrzej Nazarkiewicz, Bernd Hoppe, Anna Wasilewska

**Affiliations:** 1 Department of Pediatric Nephrology, Medical University of Bialystok, Waszyngtona Street 17, 15-274 Bialystok, Poland; 2 Department of Pediatric Surgery, Medical University of Bialystok, Bialystok, Poland; 3 Department of Pediatrics and Developmental Disorders, Medical University of Bialystok, Bialystok, Poland; 4 Department of Urology, Regional Hospital of Bialystok, Bialystok, Poland; 5 Department of Pediatrics, Division of Pediatric Nephrology, University of Bonn, Bonn, Germany

**Keywords:** Children, Citraturia, Normative values, Urolithiasis

## Abstract

**Background:**

Hypocitraturia is considered a major risk factor for calcium stone formation. However, there is no widely accepted reference database of urinary citrate excretion in children. The aim of our study was to determine the amount of citrate eliminated in the urine over a 24-h period in a pediatric cohort and to determine an optimal unit reflecting excretion.

**Methods:**

The study cohort comprised 2,334 healthy boys and girls aged 2–18 years. The levels of urinary citrate were assessed by an enzymatic method in 24-hour urine and expressed in absolute values, as urinary concentration, citrate/creatinine ratio, per kilogram of body weight, in relation to 1.73 m^2^, and as the calcium/citrate index.

**Results:**

Similar incremental age-related citraturia rates were observed in both male and female subjects until puberty during which time citrate excretion became significantly higher in girls. Urinary citrate adjusted for creatinine and for body weight showed a significantly decreasing trend with increasing age in both sexes. Urinary citrate corrected for body surface was weakly correlated with age. Thus, the assumption of 180 mg/1.73 m^2^/24 h for males and 250 mg/1.73 m^2^/24 h for females as lower cut-off values appeared to be reliable from a practical perspective.

**Conclusions:**

We found distinct sex-dependent differences in citraturia at the start of puberty, with significantly higher values of urinary citrate in girls than in boys. Further prospective studies are warranted to elucidate whether this difference represents a differentiated risk of urolithiasis.

## Introduction

Low urinary citrate excretion is recognized as a major risk factor for calcium stone formation. The inhibitory effects of citrate on crystal formation in urine are complex. Citrate creates soluble complexes with calcium, thereby effectively reducing urinary calcium supersaturation and preventing the nucleation of both calcium oxalate and calcium phosphate [1, 2]. In addition, citrate can directly inhibit calcium oxalate crystal growth, aggregation and attachment to renal epithelial cells by adsorbing to crystal surfaces [3, 4]. Citrate enhances the inhibitory effect of Tamm–Horsfall protein on calcium oxalate aggregation and may reduce the expression of urinary osteopontin, which is a common component of the urinary stone matrix [5].

Changes in urinary citrate level are predominantly influenced by the acid–base status. Both systemic and intracellular acidosis may inhibit renal citrate output if the citrate reabsorption and metabolism in the proximal tubule becomes upregulated in order to counteract those disorders. Therefore, higher urine pH tends to be associated with higher urine citrate [1, 6, 7].

Hypocitraturia is a frequent co-occurring metabolic abnormality among children and adolescents suffering from renal stones, with a reported incidence of 10–64 % [8–10]. Such high discrepancies in the incidence of hypocitraturia are due to a number of factors, including the different reference values for urinary citrate adopted by researchers and whether the examinations are provided on random or on 24-h collection urine samples. There are also inconsistencies in pediatric studies regarding the effects of age and gender on the urinary excretion of citrate. According to some investigators, the difference between boys and girls begins at puberty [11, 12] and, consequently, commonly used reference values for urinary citrate in younger children cannot be used after this age. Whereas some authors have stressed the need to establish daily total citrate excretion values in children, analogically to adult reference values, others have emphasized that the actual urinary citrate concentration might be much more important than the total 24-h urinary citrate output [13, 14].

We have hypothesized that 24-h urinary citrate excretion changes with age during the growth period and differs between the sexes following the onset of puberty. Therefore, in this study we determined (1) 24-h urinary citrate excretion in healthy pre-school- and school-children, (2) evaluated whether citrate output changes with age during growth and whether this change (if present) is sex-dependent(3) determined which units expressing urinary citrate were the most appropriate from a practical perspective.

## Methods

### Study population

This prospective cohort study was carried out in the Department of Pediatric Nephrology, University Children’s Hospital in Bialystok during the period 2006–2012. In total, 2,334 healthy Caucasian children and adolescents aged 2–18 (median 10.38) years were enrolled, with equal numbers of both sexes. A group of 1,050 subjects had taken part in our previous investigation [15]. The study population was divided into 16 age groups, consisting of 150 subjects in each 1-year group (75 boys, 75 girls) except for the youngest group (third year of life) which included 84 children (42 of each sex). The children/adolescents were mainly volunteers and had been approached while visiting the academic center for treatment of monosymptomatic primary nocturnal enuresis or inguinal hernia or were healthy children of hospital staff families. The exclusion criteria included abnormalities in dipstick urinalysis (Bayer Diagnostic, Bridgend, UK), any infections or medications, diseases known to affect urinary citrate excretion (i.e., renal tubular acidosis, chronic diarrhea, inflammatory bowel diseases), and inadequate 24-h urine collection assessed with urine creatinine excretion according to Remer et al. [16]. None of the participants reported a history of urolithiasis, and their screening based on ultrasonography excluded urinary stones and/or nephrocalcinosis. All subjects were evaluated using the same protocol, which included anthropometric measurements [weight, height, body mass index (BMI)], and were requested to continue their customary diet during the study. Both parents and children (when the latter were aged >15 years) gave informed consent for the study. The study was approved by the ethics committee of the Medical University of Bialystok. A single 24-hour urine collection was provided at home. After voiding, urine was stored in a sterile container at 4 °C without preservatives, and all measurements were conducted in hospital laboratory within 24 h of the end of the collection period.

### Urinary measurements

Urinary calcium and creatinine were measured using a Cobas-Integra 800 analyzer and Roche reagents (Roche, Indianapolis, IN). Urinary pH was determined using a microcomputer pH-meter (model CP-315 M; Elmetron, Zabrze, Poland). Urinary citrates were assessed by an enzymatic method using a commercial set (R-Biopharm AG, Darmstadt, Germany). Twenty-four-hour citrate excretion was expressed in absolute values, adjusted for urinary creatinine, per kilogram of body weight, related to the 1.73 m^2^ of body surface area, and as urinary concentration. The urinary calcium/citrate ratio was also determined as a stone risk index. Renal ultrasonography was carried out by a certified radiologist using high-resolution equipment.

### Statistical analyses

Median values of the above specified variables for the entire male and female populations and each 1-year subgroup were analyzed and reported. Additionally, for further analysis the subjects were divided into three age subgroups: 2–6.99, 7–12.99 and 13–17.99 years. Statistical analyses were performed using Statistica®, ver. 10.0 PL (StatSoft Inc, Tulsa OK). The Mann–Whitney *U* test was used for comparisons between two independent parameters, and the correlations were made with Spearman test. A *p* value of <0.05 was considered to be statistically significant.

## Results

### Comparison of results in boys and girls

The comparison of urinary excretion parameters in male and female study participants are given in Table 1. Compared to boys, the girls presented with significantly higher daily urinary citrate excretion independent of its manner of expression. The median 24-h citrate excretion was 476 and 400 mg/24 h in girls and boys, respectively. The differences between boys and girls in urinary citrate were more pronounced when adjusted for creatinine (683 and 509 mg/g creatinine/24 h in girls and boys, respectively) or per kilogram of body weight (12.84 and 10.45 mg/kg/24 h, respectively) as male subjects had a significantly higher weight and creatinine excretion. Calciuria was equal in both sexes, while median urinary pH was slightly but significantly lower in boys (6.29 vs. 6.39). As a consequence, the median urinary calcium/citrate ratio was significantly higher in boys (0.18 vs. 0.13 mg/mg).

**Table 1:** Comparison of urinary excretion parameters in male and female study participants

Demographic and urinary excretion parameters	Boys (*n* = 1,167)	Girls (*n* = 1,167)	Significance (*p*)
Age (years)	11.30 (3.43–17.31)	11.49 (3.63–17.71)	0.89
Weight (kg)	43.00 (16.00–77.00)	40.00 (16.00–64.00)	<0.001
BMI (kg/m^2^)	18.47 (14.12–24.45)	18.15 (14.12–23.68)	<0.001
Urine pH	6.29 (5.66–7.03)	6.39 (5.69–7.05)	<0.001
Calcium (mg/kg/24 h)	1.81 (0.75–3.79)	1.83 (0.73–3.41)	0.66
Creatinine (g/24 h)	0.94 (0.28–1.79)	0.72 (0.24–1.28)	<0.001
Citrates (mg/dL)	46.68 (10.87–129.59)	62.17 (18.39–196.45)	<0.001
Citrates (mg/24 h)	400.43 (102.23–1,104.19)	476.51 (141.29–1,261.36)	<0.001
Citrates (mg/g creat/24 h)	509.35 (118.88–1,341.40)	683.06 (242.22–1,671.05)	<0.001
Citrates (mg/kg/24 h)	10.45 (2.53–25.93)	12.84 (4.50–31.46)	<0.001
Citrates (mg/1.73 m^2^/24 h)	560.81 (142.70–1,346.81)	677.76 (241.72–1,590.76)	<0.001
Calcium/citrates (mg/mg)	0.18 (0.05–0.68)	0.13 (0.04–0.50)	<0.001

Values are presented as the median, with the range (5 %–95 %) given in parenthesis.

*BMI* Body mass index

### Age-related changes in urinary citrate excretion in boys and girls

**Fig. 1:**
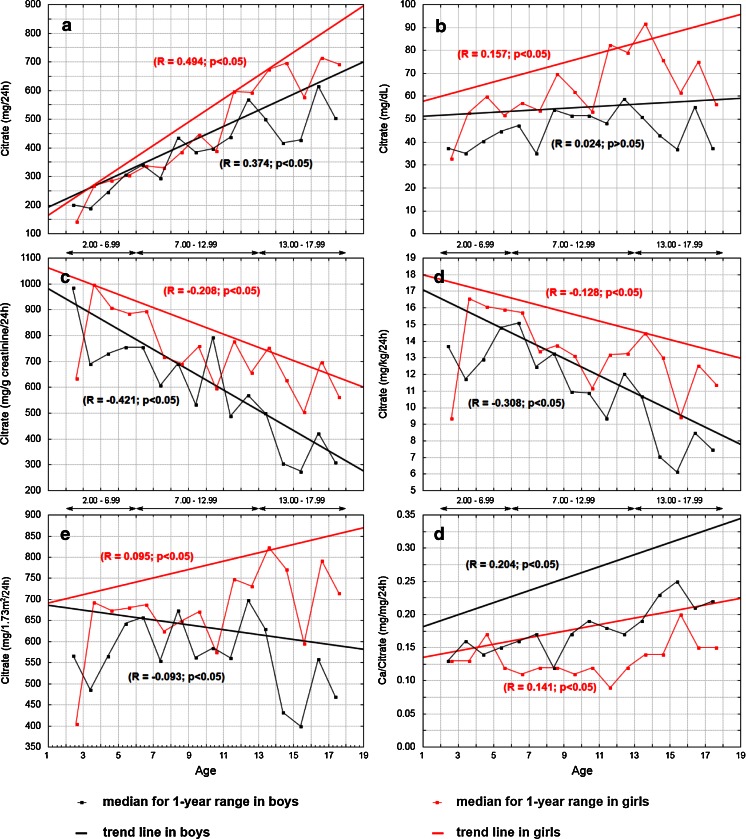
Age-dependent changes in urinary citrate excretion in healthy children and adolescents aged 2–18 years, expressed in absolute values (**a**), as urinary concentration (**b**), adjusted for creatinine (**c**), per kilogram of body weight (**d**), per 1.73 m^2^ (**e**) as the calcium/citrate ratio (**f**) and linear regression of studied variables vs. age. Linear regression values are based on trend lines

The age-related changes in urinary citrate excretion in boys and girls are shown in Fig. 1. In girls, the daily excretion rate of citrate rose from 144 mg at age 3 years to 715 mg at age 17 years; in boys, it rose from 202 mg at age 3 years to 615 mg at age 17 years. The increase in citrate output was similar in both sexes up to the age of 12 years at which time it increased sharply in girls. A similar rise in citrate output was observed in boys 1 year later, but it decreased again in the following years, resulting in urinary citrate excretion being significantly higher values in females (Fig. 1a). The median 24-h urinary citrate concentrations during all study years were higher in girls than in boys, except for the youngest children during the third year of life, when it was slightly higher in boys. The median for 24-h urinary citrate concentration rose with age, and this age-related increase was significant in girls (*R* = 0.157, *p* < 0.05), but not significant in boys (*R* = 0.024, *p* > 0.05 (Fig. 1b). Following adjustment for creatinine and for body weight, urinary citrate excretion showed yearly differences but generally presented a significant trend toward decreasing values with increasing age in both sexes. The median urinary citrate/creatinine ratio ranged from 985 mg/g at age 3 years to 275 mg/g at age 16 years in boys and from 997 mg/g at age 4 years to 504 mg/g at age 16 years in girls (Fig. 1c). The values for urinary citrate excretion expressed per 1.73 m^2^ of standard body surface area clearly differed between sexes beginning from age 14 years and were higher in female. The relationships with age displayed a weak positive correlation in girls (*R* = 0.095) and a weak negative correlation in boys (*R* = −0.093) (Fig. 1e). The urinary calcium/citrate ratio was higher in boys than in girls and exhibited trends towards increasing values with age in both genders (*R* = 0.204 and *R* = 0.141, respectively). A sharp rise in the ratio beginning from age 14 was found in boys (Fig. 1f).

### Comparison of results in boys and girls according to three age subgroups: 2–6.99, 7–12.99 and 13–17.99 years

In children aged 2–6.99 years, weight, BMI, urinary pH, creatinine, and calcium as well as the daily total excretion of citrate, citrate per kilogram of body weight and per 1.73 m^2^ did not differ significantly between sexes (Table 2). Urinary citrate adjusted for creatinine, citrate concentration and calcium/citrate ratio differed slightly but significantly between boys and girls. In the age subgroups 7–12.99 and 13–17.99 years, despite a higher body weight and BMI in boys, the urinary citrate excretion rates were higher in girls independent of its expression. Median urine pH values decreased with increasing age and additionally were lower in male participants. We did not find significant differences in either urinary pH or citrate excretion between male and female participants aged 2–6.99 years, whereas in older male subgroups, the urinary pH and citrate excretion were significantly lower than those in girls (Table 2).

**Table 2:** Comparison of urinary parameters in male and female study participants stratified into three age groups

Urinary parameters	Study participants		Significance (*p*)
Age group: 2–6.99 years			
	Boys (*n* = 342)	Girls (*n* = 342)	
Age (years)	4.77 (2.47–6.71)	4.78 (2.75–6.66)	0.23
Weight (kg)	18.50 (13.50–28.00)	18.50 (13.55–25.00)	0.25
BMI (kg/m^2^)	15.61 (13.50–18.89)	15.44 (13.15–18.18)	0.37
Urine pH	6.52 (5.71–7.16)	6.49 (5.69–7.30)	0.73
Calcium (mg/kg/24 h)	1.87 (0.88–3.72)	1.90 (0.76–3.29)	0.71
Creatinine (g/24 h)	0.34 (0.18–0.51)	0.33 (0.22–0.44)	0.51
Citrates (mg/dL)	40.99 (11.21–127.69)	52.09 (15.22–135.34)	<0.001
Citrates (mg/24 h)	258.19 (67.14–659.06	278.77 (87.17–734.13	0.14
Citrates (mg/g creat/24 h)	763.45 (242.69–1,778.37)	863.19 (290.36–2,369.60)	0.04
Citrates (mg/kg/24 h)	13.59 (4.21–3.90)	15.06 (5.22–41.40)	0.06
Citrates (mg/1.73 m^2^/24 h)	591.15 (176.97–1,468.67)	650.56 (218.58–1,726.39)	0.09
Calcium/citrates (mg/mg)	0.15 (0.05–0.48)	0.13 (0.03–0.42)	<0.001
Age group: 7–12.99 years			
	Boys (*n* = 450)	Girls (*n* = 450)	
Age (years)	10.38 (7.21–12.72)	10.31 (7.27–12.75)	0.33
Weight (kg)	36.00 (23.00–57.00)	35.00 (22.40–53.00)	0.02
BMI (kg/m^2^)	17.72 (14.26–23.51)	17.36 (14.13–22.49)	0.09
Urine pH	6.22 (5.69–7.01)	6.40 (5.71–7.00)	<0.001
Calcium (mg/kg/24 h)	1.91 (0.79–3.75)	1.56 (0.55–3.36)	<0.001
Creatinine (g/24 h)	0.80 (0.50–1.12)	0.66 (0.44–0.99)	<0.001
Citrates (mg/dL)	50.06 (11.28–127.41)	64.19 (18.81–210.35)	<0.001
Citrates (mg/24 h)	425.14 (102.26–989.02	446.11 (161.63–1,067.23	0.003
Citrates (mg/g creat/24 h)	550.38 (139.03–1,212.10)	687.29 (261.44–1,462.17)	<0.001
Citrates (mg/kg/24 h)	11.35 (3.13–24.81)	12.76 (4.98–29.92)	<0.001
Citrates (mg/1.73 m^2^/24 h)	599.90 (162.50–1,272.99)	656.66 (253.03–1,498.15)	<0.001
Calcium/citrates (mg/mg)	0.17 (0.05–0.60)	0.12 (0.04–0.47)	<0.001
Age group:13.00–17.99 years			
	Boys (*n* = 375)	Girls (*n* = 375)	
Age (years)	15.44 (13.26–17.82)	15.34 (13.31–17.88)	0.64
Weight (kg)	65.20 (42.00–81.90)	55.00 (40.00–69.00)	<0.001
BMI (kg/m^2^)	21.13 (16.44–25.25)	20.45 (17.22–24.24)	<0.001
Urine pH	6.22 (5.63–6.90)	6.35 (5.66–6.96)	<0.001
Calcium (mg/kg/24 h)	1.71 (0.71–3.79)	1.93 (0.92–3.50)	<0.001
Creatinine (g/24 h)	1.40 (0.97–1.94)	1.08 (0.80–1.36)	<0.001
Citrates (mg/dL)	43.96 (10.11–142.79)	70.77 (21.30–211.46)	<0.001
Citrates (mg/24 h)	490.12 (130.05–1,327.90)	659.58 (230.55–1,589.42)	<0.001
Citrates (mg/g creat/24 h)	339.65 (89.68–976.42)	610.83 (216.30–1,403.67)	<0.001
Citrates (mg/kg/24 h)	7.69 (2.17–21.98)	12.28 (4.16–28.38)	<0.001
Citrates (mg/1.73 m^2^/24 h)	475.97 (130.82–1,329.07)	729.45 (248.75–1,696.49)	<0.001
Calcium/citrates (mg/mg)	0.22 (0.06–0.80)	0.16 (0.05–0.60)	<0.001

Values are presented as the median, with the range (5–95 %) given in parenthesis.

BMI, Body mass index

Table 3 provides the 5th, 10th, 25th and 50th percentiles for urinary citrate excretion in children with stratification into three age subgroups, namely, 2–6.99, 7–12.99 and 13–17.99 years.

**Table 3:** Values of 5th, 10th, 25th and 50th percentiles for 24-h urinary citrate excretion for boys and girls with stratification into three age subgroups

Urinary citrate excretion parameters	Boys				Girls			
	5 %	10 %	25 %	50 %	5 %	10 %	25 %	50 %
Age group: 2–6.99 years								
Citrates (mg/dL)	11.2	14	26	41	15.2	20	30	52
Citrates (mg/24 h)	67.1	108	168	259	87.2	115	169	279
Citrates (mg/g creatinine/24 h)	242	329	516	763	290	365	558	863
Citrates (mg/kg/24 h)	4.2	6.1	9.3	13.5	5.2	6.8	9.6	15,1
Citrates (mg/1.73 m^2^/24 h)	177	264	401	591	218	284	408	651
Age group: 7–12.99 years								
Citrates (mg/dL)	11.3	16	29	50	18.8	25	38	64
Citrates (mg/24 h)	102	161	280	425	161	213	308	446
Citrates (mg/g creatinine/24 h)	139	220	360	550	261	341	490	687
Citrates (mg/kg/24 h)	3.1	4.5	7.4	11.3	5.0	6.4	9.2	12.8
Citrates (mg/1.73 m^2^/24 h)	162	240	400	600	253	331	481	657
Age group: 13–17.99 years								
Citrates (mg/dL)	10.1	15	27	44	21.3	28	44	70
Citrates (mg/24 h)	130	206	323	490	230	313	444	660
Citrates (mg/g creatinine/24 h)	89	144	231	340	216	275	430	611
Citrates (mg/kg/24 h)	2.2	3.2	5.2	7.7	4.2	5.3	8.3	12.3
Citrates (mg/1.73 m^2^/24 h)	130	200	327	476	248	328	486	729

## Discussion

All children with renal stones and nephrocalcinosis should undergo a metabolic evaluation to identify the etiology of the disease. Several recent studies have indicated hypocitraturia as one of prevalent and most important causative factors of these conditions [9, 10, 17, 18]. However, normative data on urinary citrate excretion during development in terms of clinical validity, the units used and possible changes with age and gender distinction are not widely acceptable. Hypocitraturia in children has been defined as 24-h citrate excretion of <400 mg/g creatinine [19] or <180 mg/g creatinine regardless of gender [20]. Other definitions include sex-dependent differences and consider the lower limits to be a daily urine citrate of 125 mg/g creatinine in boys and 300 mg/g creatinine in girls [11] or 1.9 mmol (365 mg)/1.73 m^2^ in males and 1.6 mmol (310 mg)/1.73 m^2^ in females [21].

In our study, we sought to determine the age and sex dependence of urinary citrate excretion in healthy individuals. Total citrate voiding rates exhibited a similar yearly increments in boys and girls up to the age of 11 years, reflecting increased food consumption, muscle mass and body size as children become older. Girls at age 12 years and boys at approximately age 13 years, ages that correspond to the onset of puberty in girls and boys, respectively, showed sharp increases in citrate output. Afterwards, between ages 14 and 18 years the daily total citrate excretion was already significantly higher in girls. Our results suggest that female sex hormones may play an important role in determining the urinary citrate level. Other studies conducted on premenopausal and menopausal women to assess the influence of estrogen on urinary chemical parameters showed that citrate excretion varied during the menstrual cycle and was higher during the high compared to the low basal body temperature phases, gradually falling in menopausal women [22, 23]. The distinction in urinary citrate excretion between the sexes can also be partially explained by ascertained differences in urinary pH. In our study, median urinary pH values decreased with increasing age and additionally were lower in male than in female participants—these changes were in similar directions to those observed for citrate when calculated in relation to weight or creatinine excretion. We can speculate that the fall in urinary pH with age is due to changes in dietary habits and exertion.

The reference values for urinary citrate which have hitherto been used in children are associated with urinary creatinine [11, 19, 20] or to the 1.73 m^2^ of standard body surface area [21]. In our study, the median of urinary citrate/creatinine ratio was higher in girls than in boys (683 vs. 509 mg/g) and showed a significant trend to decrease with increasing age in both sexes (*R* = −0.208 and *R* = −0.421), respectively. The 5th percentile of the ratio was 170 mg/g for the entire study population and was similar to that found by Srivastava et al. [20], whereas it amounted to 118 and 242 mg/g in boys and girls, respectively. Thus, adoption of a single definition of hypocitraturia (i.e., <180 mg/g creatinine or <400 mg/g creatinine) for both sexes and among different age groups within the pediatric population appears to be problematic. Because of the relevant age and sex dependence of citraturia observed in our study, the definition of hypocitraturia when corrected for creatinine should be established based on normative data for both sexes and for separate age periods up until adulthood. Similar observations with regard to the urinary citrate/creatinine ratio in children were reported by Borawski et al. who proposed stratification of urinary reference values for citraturia according to quintiles of age [24].

We found that the threshold of lower normal limits (5th percentile) for the citrate/creatinine ratio were distinctly different for boys and girls and also age dependent, being 242 and 290 mg/g, respectively, in the subgroup aged 2–6.99 years, 139 and 261 mg/g, respectively, in the subgroup aged 7–12.99 years, and 89 and 216 mg/g, respectively, in the subgroup aged 13–17.99 years. The lower citrate level and especially the higher creatinine excretion in adolescent males compared to girls seems to be the explanation for an increasing sex-related divergence in the citrate/creatinine ratio within these age ranges.

In our study, urinary citrate related to 1.73 m^2^ of standard body surface area presented as approximate median values in boys and girls for subgroups aged 2–6.99 and 7–12.99 years (591 vs. 650 and 599 vs. 656 mg/1.73 m^2^, respectively). The median value in boys was significantly lower than that in girls in the subgroup aged 13–17.99 years (475 vs. 729 mg/1.73 m^2^, respectively). This result is important because this manner of expression exhibited much less variability than the other ‘units’ within the groups of boys and girls, respectively, and there was only a weak positive correlation with age in girls (*r* = 0.095) and a weak inverse trend with age in boys (*r* = −0.093). The 5th percentiles established for the three age subgroups were at 177, 162 and 130 mg/1.73 m^2^ (in increasing age subgroups) for boys and 218, 253 and 248 mg/1.73 m^2^ (in increasing age subgroups) for girls. Therefore, from a practical perspective, we recommend simplifying the reference values for urinary citrate expression in children of all ages and to use 180 mg/1.73 m^2^/24 h in males and 250 mg/1.73 m^2^/24 h in females for hypocitraturia screening.

However, we should be aware that urinary citrate levels that fall below the 5 % of reference range suggest a need for investigation in terms of possible metabolic acidosis (e.g., renal tubular acidosis) [11, 13]. Additionally, the optimal urinary citrate excretion for calcium stone formers should be closer to the statistical average than to the lower limits of the healthy reference group, and any value lower than the mean/median for 24 h may still represent a potential risk for kidney stone formation or recurrence, particularly in patients with coincident hypercalciuria [14, 20]. Therefore, we show here the 5th, 10th, 25th, and 50th percentiles for urinary citrate expressed using different units for three age periods, as these may also be applicable in clinical practice, such as for pediatric stone formers treated with citrate supplements (Table 3).

Many authors have postulated that not only the absolute urinary calcium or citrate concentrations, but alternatively the relative proportion of the two, determine the risk of stone formation [20, 25, 26]. It has been shown that a urinary calcium/citrate index of 0.326 mg/mg can discriminate well between pediatric stone formers and healthy controls [20]. In this study, the medians of the above quotient were below that value in the all-age group. Nevertheless, the age-related continuous increase in the urinary calcium/citrate ratio, particularly in boys aged >14 years, was detected in our study. This may partly imply a higher prevalence of urolithiasis in older individuals and also a higher susceptibility to kidney stone disease among males.

There are a few limitations to our study. The most important weakness is perhaps that we have not extended our investigation to include infants and children aged ≤2 years who were not toilet-trained. Furthermore, the subgroup of children in their third year of life was quite small compared to the other age groups, and the results in this group did not match general trends of our findings. Finally, our study cohort is homogeneous, and other populations with more varied racial and ethnic groups may be needed for validation of our results over time.

Our results, based on a large representative population of healthy children and adolescents, may provide useful normative data to screen for urinary citrate, particularly in individuals at risk of urolithiasis and/or nephrocalcinosis. In contrast to other available reference values in children, this report shows specific and more precise cut-off points in citrate excretion by indicating differences between boys and girls in critical stages of growth. The determination of a distinct urinary citrate reference may be of value in monitoring management with citrate supplements in pediatric stone disease.
